# OmniMatch: A Large Language Model-Based Data Linkage Tool

**DOI:** 10.23889/ijpds.v9i5.2588

**Published:** 2024-09-18

**Authors:** Xiaowei Xu, Xingqiao Wang, Vivek Gunasekaran, Jonathan White, Anup Mathur

**Affiliations:** 1 University of Arkansas at Little Rock; 2 US Census Bureau

Large Language Models, such as OpenAI’s GPT, have demonstrated remarkable success in various applications by generating human-like text. However, a critical question remains: how can we effectively leverage these large language models for data linkage? In response to this challenge, we introduce OmniMatch, a novel data linkage tool designed to address several key issues in data linkage:

- Cross-Domain Data Linkage: OmniMatch tackles the complexities of linking data across different domains without retraining or update.
- Cross-Lingual Data Linkage: It extends its capabilities to handle data in multiple languages and hybrid languages.
- Data Quality Challenges: OmniMatch addresses inconsistent data formats and typical data quality issues, including noise, missing values, typos, and errors.

Our approach customizes an open-source large language model called Llama 2 from Meta. By doing so, we achieve outstanding performance in handling the aforementioned challenges. Notably, OmniMatch offers a specific advantage: it can be installed on-premises, ensuring a safe and trustworthy application without the hallucinations and other vulnerabilities associated with foundational large language models.

We systematically evaluate OmniMatch using diverse datasets from various domains, including products, scientific publications, music, census data, and cross-lingual data. The experimental results demonstrate that OmniMatch is a universally applicable and trustworthy tool for data linkage.

